# P-1881. Biopreparedness and Biocontainment Education in U.S. ID Fellowships

**DOI:** 10.1093/ofid/ofaf695.2050

**Published:** 2026-01-11

**Authors:** Cristina J Torres, Angela Hewlett, James Lawler, Daniel Cybulski, Gavin Harris, Elizabeth Schnaubelt, David Brett-Major

**Affiliations:** University of Nebraska Medical Center, Omaha, Nebraska; University of Nebraska Medical Center, Omaha, Nebraska; University of Nebraska Medical Center, Omaha, Nebraska; University of Nebraska Medical Center, Omaha, Nebraska; Emory University, Atlanta, Georgia; University of Nebraska Medical Center, Omaha, Nebraska; University of Nebraska Medical Center, Omaha, Nebraska

## Abstract

**Background:**

Infectious Disease (ID) physicians play a key role in public health emergencies and communicable disease threats. Their involvement spans several domains: patient care (including development and deployment of medical countermeasures), design and conduct of research, implementation of prevention and control measures, and coordination with public health officials. We sought to inventory the existing biopreparedness and biocontainment training offered in ID fellowship training programs (FTP) in the United States to identify gaps and inform the development of resources to address them, ultimately strengthening workforce development in this critical area.

**Figure:**
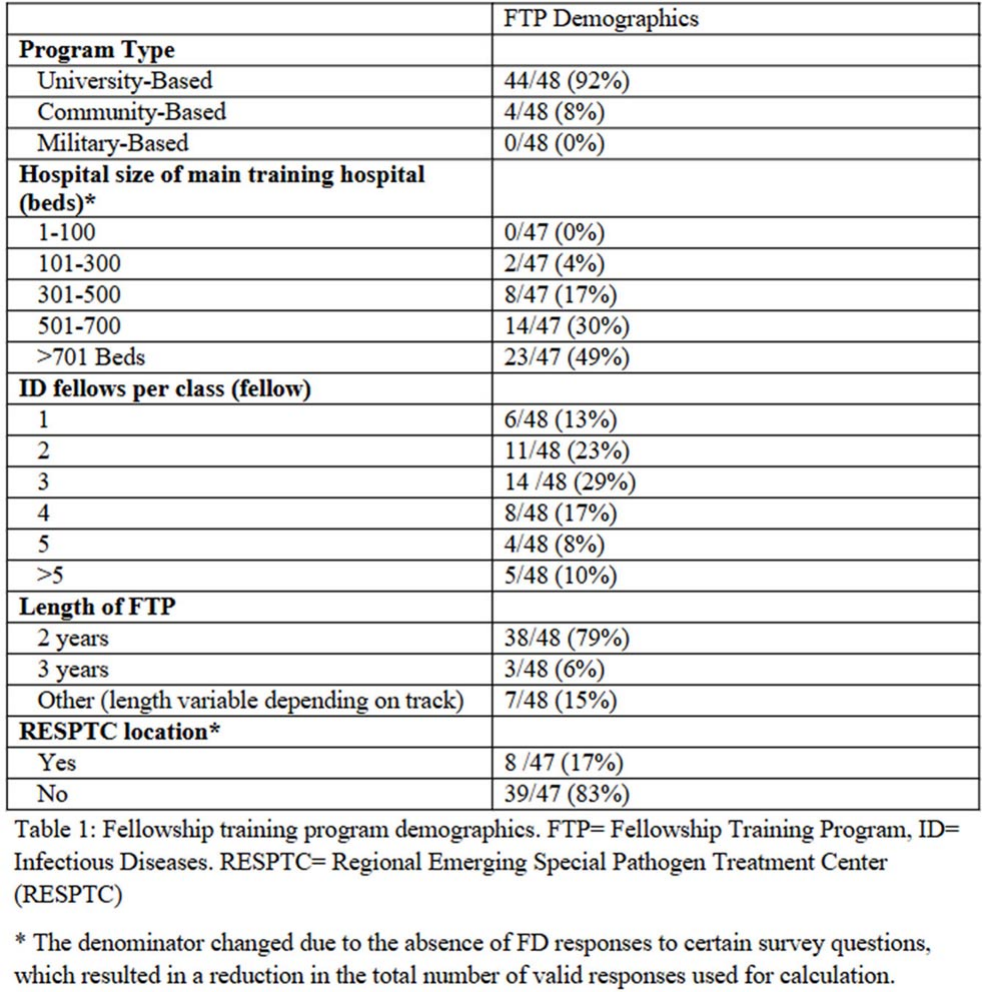

**Figure:**
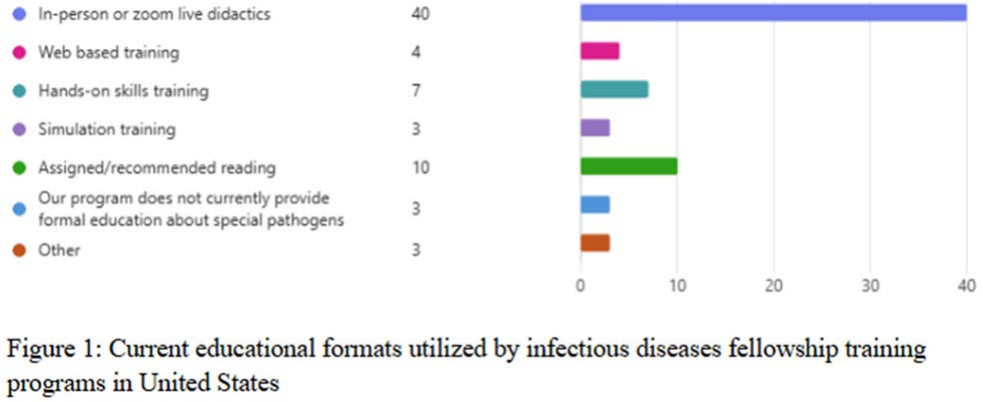

**Methods:**

This study employed an anonymous, cross-sectional, online survey directed at ID fellowship directors (FD) in the United States. A total of 166 ID FTPs were identified through the American Medical Association’s Fellowship and Residency Electronic Interactive Database (FREIDA), and FD contact emails were manually extracted and verified through individual program websites. Responses were collected electronically and incorporated into gap analysis.

**Figure:**
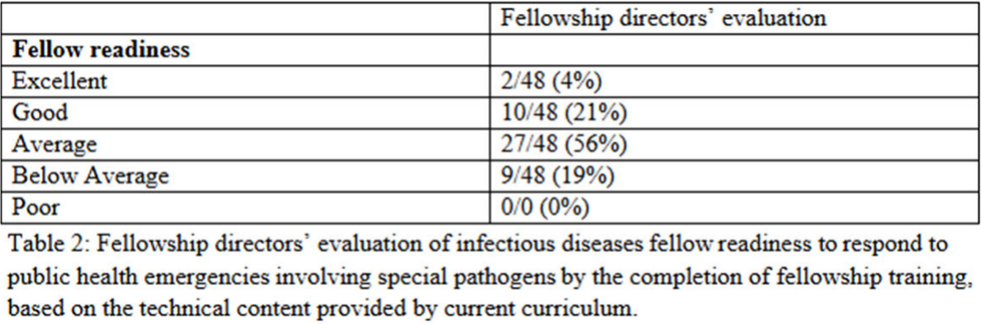

**Figure:**
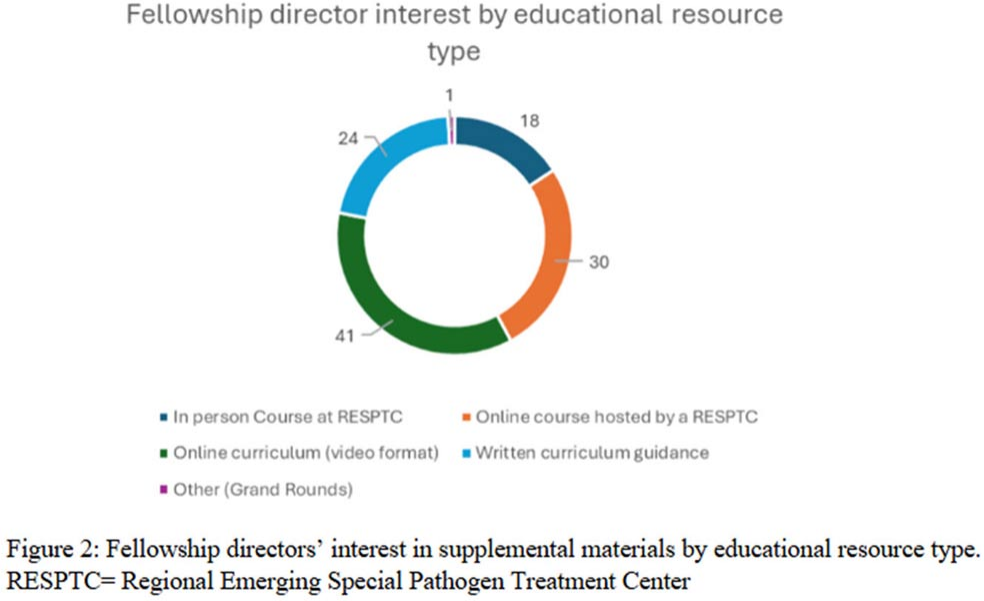

**Results:**

Between July and October 2024, 48 ID FDs completed the survey, yielding a 29% response rate. Demographic characteristics of the participating programs are presented in Table 1. Of the respondents, 8 (17%) identified their associated hospitals as Regional Emerging Special Pathogen Treatment Centers (RESPTCs). 83% (n=40) of respondents reported using in-person or live Zoom didactics as their educational format (Figure 1). The majority of respondents (75%, n=36) rated their graduating fellows as having either ‘average’ or ‘below average’ readiness to respond to public health emergencies involving special pathogens (Table 2). Furthermore, 100% of respondents expressed interest in at least one new educational resource format to incorporate into their programs (Figure 2).

**Conclusion:**

There are clear opportunities to address gaps in current biopreparedness and biocontainment education and training of ID fellows. Both virtual and in-person curriculum options are needed. Further assessment of community-based training program needs is indicated.

**Disclosures:**

Angela Hewlett, MD, MS, Forecast Orthopedics: Advisor/Consultant|Mapp Biopharmaceutical Inc.: Grant/Research Support James Lawler, MD, MPH, FIDSA, Arkansas Hospital Association: Honoraria|Seqirus-CSL: Advisor/Consultant David Brett-Major, MD, MPH, Shoreland: Advisor/Consultant

